# Making a Case for “Education for Health Literacy”: An International Perspective

**DOI:** 10.3390/ijerph17041436

**Published:** 2020-02-24

**Authors:** Sandra Vamos, Orkan Okan, Tetine Sentell, Irving Rootman

**Affiliations:** 1 School of Interdisciplinary Health Programs, Western Michigan University, Kalamazoo, MI 49001, USA; 2 Interdisciplinary Centre for Health Literacy Research, Bielefeld University, 33615 Bielefeld, Germany; orkan.okan@uni-bielefeld.de; 3 Office of Public Health Studies, University of Hawai‘i at Mānoa, Honolulu, HI 96822, USA; tsentell@hawaii.edu; 4 School of Public Health & Social Policy, University of Victoria, Victoria, BC VBW 2Y2, Canada; irootman@telus.net

**Keywords:** health literacy, education, equity, health care, life course

## Abstract

In many countries, health literacy research, practice, and policy have been moving away from a focus only on medical care and health-care settings to a much broader conceptualization. In this broader perspective, health literacy can be obtained and used across many other settings (e.g., school, home, workplaces, government) towards achieving health and wellness goals across the life-course for individuals, families, and communities. The education sector is a critical domain towards these achievements and *education for health literacy* is a fundamental process and outcome. This can help towards important public health goals, including critical health literacy, as oriented not only towards individual actions, but also towards supporting effective social and political action. This Perspective Article describes the importance and utility of the *education for health literacy* perspective, which, follows a view that health literacy is a *key outcome of health education* from which improved population health, health promotion and disease prevention could be achieved across diverse contexts. We first describe different educational paradigms to address health literacy and clarify the *education for health literacy* perspective as a supportive, instructional and capacity-building global resource across the life-course. Then, using specific examples from Canada, America, and Germany, we provide a snapshot of the diverse ways in which the *education for health literacy* perspective can be found in national policies. These include broad national goals and standards (Germany and Canada) and major health care reform (America). We next consider the tensions and gaps that can arise in the translation and implementation of these policies relative to the ideal education for health literacy perspective, especially related to equity. These include the need for funding, goals of the educational system, and limited evaluation of policy in practice. Finally, we highlight strategic opportunities to achieve education for health literacy and equity especially offering examples from innovative practice in Canada across the lifespan.

## 1. Introduction

Health literacy is a growing research and practice field concerned with the capacities of people to meet the complex demands of health across the life-course in our modern society [1,2]. Existing definitions of health literacy take into account how people find, understand, evaluate, use, and communicate health information [3]. Another way of conceptualizing health literacy is to categorize the capabilities into basic/functional literacy skills, communicative/interactive, and critical literacy skills [4]. The latter allows individuals to appraise health information and direct individual, social, and political action towards collective wellbeing [2,4]. Health literacy can be achieved and used across many settings (e.g., school, home, workplaces, government) towards achieving health and wellness for individuals, families, communities, and nations [5].

Strong evidence exists that health literacy skills (across a variety of definitions and measurement tools) are limited even in wealthy, developed countries [6]. Findings from the first European comparative survey on health literacy in populations [7], which was conducted in eight countries, found almost half of the respondents (47%) had limited (insufficient or problematic health literacy). Similar results have been shown in earlier studies from the United States (US) and Canada. The survey conducted by the former Canadian Council on Learning [8], estimated that about 2/3 Canadian adults and 9/10 of seniors lacked the capacity to obtain, understand and act on health information and services and make appropriate health decisions on their own. According to the US Department of Health and Human Services [9], 9 out of 10 adults have difficulty using the everyday health information that is routinely available to them in different settings in their daily lives.

Compared to adult health literacy, evidence on children and adolescents’ health literacy is rather limited. The Finnish data from the Health Behavior in School-aged Children survey (HBSC) shows that 1/3 of the 7th and 9th graders had high levels of health literacy and around 60% had shown moderate levels [10]. The HBSC results for Lithuania show high levels of health literacy in 17.4% of the children, while 70.5% were found to have moderate levels [11]. A school-based survey on health literacy in 6th graders in Taiwan found that 44% of the children had high levels of health literacy (high: 16.3%; very high: 27.7%), while 56% had low health literacy (very low: 26.6%; low: 29.5%) [12]. In the past, studies have also shown that higher levels of health literacy are associated with better health behaviors in adolescents [13], that higher health literacy in school-aged children is associated with increased health outcomes [10,12] and that higher levels of health literacy have an impact of physical activity and self-reported health in adolescents [14].

Importantly, across populations within these countries, there are also significant health literacy inequities [7,15,16]. In the US, racial and ethnic minorities, those who do not speak English, those with low income and/or low education, those who are older, and those who live in rural communities are all disproportionately likely to have low health literacy [17]. Certain segments in Canadian society are similarly disproportionately affected by low health literacy, especially Aboriginal populations, older adults, recent immigrants, people with low levels of education, those with low English/French proficiency, and those on social assistance [18,19]. In context of these inequities and health disparities, health literacy is considered a modifiable and intermediary factor that can be changed by educational means [20,21,22,23].

Education is one of the key settings identified as a common denominator to address poor health literacy around the globe [24]. Drawing on the 1986 *Ottawa Charter for Health Promotion* [25] to the 2016 *Shanghai Declaration on Health Promotion* [26], many scholars and practitioners around the world have been making a case for the important role that education can play with other sectors to enhance health literacy. The education sector presents many important settings to address the promotion of health literacy. Schools can reach all school-aged children over a long period of time [27] making them a perfect target for long time interventions to develop and strengthen health literacy capacities [28,29,30]. In schools, children from different socio-economic and cultural backgrounds can be reached, in theory, ensuring that all receive equal support, attention and opportunity, or such that is proportionate to the degree of their disadvantage, to develop health literacy [5,31,32,33]. In the context of sustainable health promotion action, the education sector offers several whole-school approaches addressing various environmental dimensions affecting health and development [5,32]. These include programs that support nutrition towards goals of better outcomes in health and academic achievement [34,35]. In terms of sustainability and cost effectiveness, school-based health literacy programs are understood to be promising initiatives based on an evidence synthesis regarding the benefits of early health literacy interventions in the education sector [36].

This Perspective Article describes the importance and utility of the *education for health literacy* perspective, which follows a view that health literacy is a *key outcome of health education* from, which improved population health, health promotion and disease prevention could be achieved across diverse contexts. While the term “education” is broad, our article focuses specifically on the formal education sector for children and youth and what it can do to promote the educational paradigm of *‘education for health literacy,’* which in turn supports the role of the health care sector and other partners. We focus on this aspect of education as childhood and youth are critical time periods for the development of skills, behaviors, knowledge, and social-emotional learning that can lead to health literacy over the lifespan [37].

We first describe different educational paradigms to address health literacy and clarify the *education for health literacy* perspective, which we consider as a supportive, instructional and capacity-building global resource across the life-course and describe different educational paradigms. Then, using specific examples from Canada, America, and Germany, we provide a snapshot of the diverse ways in which the *education for health literacy* perspective can be found in national health literacy policies. This provides a useful landscaping of current policy activity relevant to this perspective and also identifies potentially fruitful areas for future policy implementation and evaluation research. Towards this goal, we consider the tensions and gaps that can arise in the translation and implementation of these policies relative to the ideal education for a health literacy perspective, especially related to equity. Finally, we highlight strategic opportunities to achieve education for health literacy and equity using examples from innovative practice in Canada across the lifespan. We thus consider how the growing awareness and potential implications of educational priorities translates into meaningful collective and concrete actions.

## 2. Educational Paradigms

Interest in the evolving concept of health literacy around the globe has developed from three main perspectives: (1) health care; (2) health promotion; and (3) education [38]. This section highlights the perspective related to education for health literacy. Paradigms or patterns can be seen within this perspective related to the relationship between health and education. The ‘education-health’ and ‘health-education’ relationship is complex. Queries and foundational assumptions have been made over the years related to common notions. However, it does not appear that the angles considering educational paradigms to address health literacy have been compartmentalized, outlined and/or compared in the literature to showcase the progression over time. We have categorized these as follows: (i) ‘no education without health’; (ii) ‘no health without education’; (iii) and/or ‘no education and health without health literacy’.

### 2.1. No Education Without Health

The important link between learning and health has existed for quite some time. The original 1918 Cardinal Principles of Secondary Education indicated that the health of the individual was essential to the nation’s vitality [39]. “A student who is not healthy, who suffers from an undetected vision or hearing deficit, or who is hungry, or who is impaired by drugs or alcohol, is not a student who will profit optimally from the educational process” [40] (p. 13). Today, this notion of ‘no education without health’ is at the forefront of only some educational planning and policy. For instance, there are innovative policies around nutrition and access to school lunch, even in the summer, for low income children that are relevant to this perspective. The idea is that nutrition-related policies can support health and academic achievement, both of which will have immediate benefits in terms of child wellbeing (e.g., children who are not hungry can pay attention, better nutritional intake reduces risk of obesity) and lead to future health-related rewards (e.g., higher educational attainment is associated with better jobs and better health) [34,35].

Some have argued that the US K-12 public education has focused on standardized test scores on reading and math to evaluate schools, teachers and students through the No Child Left Behind (NCLB) Act [41] and the Every Student Succeeds Act (ESSA) as the main federal laws for annual testing. Since 2002, these laws have shaped and defined how success is determined for students, teachers, and schools. Defining student success by how well they ‘perform on tests’ and teachers’ success as ‘teaching to the test’ vs. ‘teaching to the skills’ is a limited perspective of the Cardinal Principles and how students may “profit optimally” from education. While the nutrition-related policies noted above may improve test performance, ideal goals are larger than this. A broader premise of healthy schools and a wide range of educational activities are needed to be considered to enhance children’s and youth’s health literacy ‘skills’ for our modern society. Thus, we conclude that while the no education without health perspective is important, it is not the only way to consider the combined goals of the education and health sectors.

### 2.2. No Health Without Education

There are many models that help explain the why and how learning influences our health [42]. According to the 2008 Canadian Council on Learning [8] report, education can have a direct effect on our health by influencing a range of competencies such our ability to gather and interpret health-related information influencing coping abilities and decision-making. As the nutrition efforts mentioned above, such education skills over time can also have an indirect effect by influencing employment and income levels. The relationship of health by educational attainment is strong and pervasive across many contexts and outcomes.

Related to health literacy, educational attainment is one of the topic two factors predicting heath literacy in those ages 16–65+ [8]. For instance, in the National Assessment of Adult Literacy in the U.S., 49% of adults who did not complete high school had the lowest level of health literacy compared to 15% who had a high school diploma as their final degree and 3% of those with a bachelor’s degree [43]. Many of factors and variables can influence and contribute to the development of health literacy needed to improve positive health outcomes. Uncovering these relationships are important areas for future study, and may provide insight to health disparities [44]. However, these are often academic and theoretical discussions. They may not provide full insights into the practical skills and relationships needed to fully support health in a practical setting, especially for children who are in school today. Thus, we consider a final perspective.

### 2.3. No Education and Health Without Health Literacy

Today, there is a growing recognition internationally that health literacy starts at a very early age [29], while schools are seen promising intervention settings [26,28,29] and teachers, principals and caregivers play a key role [45,46,47]. Outside the educational sector family homes are highlighted as critical settings for health literacy development of children. The health literacy of caregivers and parents may have an impact on the development of health literacy competencies, decision-making skills, health knowledge, health behaviors and health-related beliefs [46].This is due in part to the gradual increase in reports and publications related to the importance of children and youth’s health literacy, which occurs in broader context. For example, the 2016 *WHO Policy Brief: Investing in Health Literacy* [36] makes a case for investing in school health literacy with a focus on health literacy actions to influence both educational and health outcomes, which in turn will influence future generations. In a recent systematic review of definitions and models of health literacy in childhood and youth, 9 of 21 models identified were developed from a school health education perspective [35]. This typically occurs in the education sector through health education classes and embedding health literacy within educational activities throughout the school [36].

Health literacy is a powerful bridging concept between education and health. Health literacy is described as a concept, a process, an outcome and a public health goal. We know that lower health literacy is associated with poorer health outcomes and health behaviors [9,19]. Over the years research has shown that people with limited health literacy (i.e., deficits) use preventive health services less (e.g., flu shots, mammograms, prenatal care); are unable to manage chronic conditions (e.g., high blood pressure, diabetes, asthma); are less likely to follow provider orders (e.g., use medication properly); experience negative psychological effects (e.g., shame about low skill levels); have reduced capacity to act on health alerts (e.g., severe weather, food advisories); and are more likely to self-report their health as fair or poor [9]. With this in mind, health education is a tool, that helps to improve health literacy. According to the WHO Health Promotion Glossary [48] (p. 10): “Health education comprises consciously constructed opportunities for learning involving some form of communication designed to improve health literacy, including improving knowledge, and developing life skills which are conducive to individual and community health.” A recent article by Nutbeam [4] entitled “Health Education and Health Promotion Revisited”, articulates how the concept of health literacy has been useful in the evolution of health education content and approaches. Health literacy involves “an observable set of skills that can be developed and improved through effective communication and education” [4] (p. 708). These transferable skills are assets and are continuously developed and enhanced over the life-course to adapt to the different and changing environments, contexts, and the sophistication of modern times. We need more tailored approaches and services to facilitate the acquisition of health literacy skills for different groups. Quality education and life-long learning are instrumental in the process.

The education perspective has been more prominent in some countries more than others. As one example, a glimpse into Canada’s health literacy history reveals the Federal Government declared literacy a federal priority in 1986 [49]. This started the national health literacy pathway with reports and efforts to explore the relationship between literacy and health and bringing people from both of these fields bridging the education and health sectors [49]. This perspective views health literacy as an essential bridge across professions and jurisdictions [19]. As a dynamic field, it is important to continue the education dialogue to further develop knowledge, raise awareness and build capacity, and develop partnerships and infrastructure that will continue the advancement of the international agenda. If our shared practical vision for a health literate world is to be realized, education for health literacy should be prioritized in the wider context of many stakeholders towards concrete actions to improve health and involve individuals, providers and systems [6].

## 3. Policy

This section provides snapshot of diverse ways for the *education for health literacy* perspective can be found in policy. This provides a useful landscaping of current policy activity in three countries. These may inform advocacy in other settings and point to fruitful areas for future policy implementation and evaluation research. It is first important to distinguish how countries, organizations and individuals may articulate what is a health literacy policy. An evident challenge to this goal is the reality that ‘there is not a clear consensus concerning what is and what is not a health literacy project or policy’ [50] (p. 9). Moreover, there are differences in scope in nature and extent of policies (e.g., proposed by government vs. organizations), strategies, laws, and standards. For example, while some countries have specific, binding governmental health literacy policies, other countries have health literacy strategies that are not endorsed by the governmental bodies. For this paper, we choose a broad definition of policy by Buse and colleagues describing a policy as a ”broad statement of goals, objectives and means that create the framework for activity. It often takes the form of explicit written documents, but may also be implicit or unwritten” [51] (p. 5). According to Nutbeam [4], with the burst in the number of research papers on the subject over the past decade, health literacy has become a very popular issue with governments in many countries adopting national strategies to improve health literacy.

We will share some important examples below and further analyze commonalities and differences with respect to the *education for health literacy* perspective to utilize existing and/or inform policies. Below we provide national policy examples from Canada, the United States and Germany, while there are to date no implementation and evaluation studies available on the effects of these policies, which is true for many national, regional and local policies on health literacy [52]. Opportunities and gaps will be identified later in the paper. For some basic K-12 educational context of the three international school system structures and their policies of relevance, we provide Table 1 further below.

### 3.1. Policy in Canada

In Canada, the health literacy pathway is anchored in broad education and health promotion perspectives. Pockets of efforts and expertise across the country have been informed by noteworthy international landmark policy documents, such as the *Ottawa Charter for Health Promotion* [25]. However, there is a lack of a ‘formal’ national health literacy policy to inform Canadian work. International adult literacy surveys [53,54] and Canada’s own production of significant reports, products and resources reinforce the need for official policy to underpin practice. The recent book chapter entitled Health Literacy Policies: National Examples in Canada by Vamos and colleagues [49], highlighted the two key landmark national documents that have informed Canadian perspectives, practice, and policy. First, in 2008 the Canadian Expert Panel on Health Literacy (Expert Panel) produced *A Vision for a Health Literate Canada* report with a vision statement that: ‘All people in Canada have the capacity, opportunities and support they need to obtain and use health information effectively, to act as informed partners in caring for themselves, their families and communities, and to manage interactions in a variety of settings that affect health and well-being’ [18] (p. 23). The Expert Panel recommended a national strategy with policies to increase the levels of health literacy of all Canadians and decrease health disparities. However, no national strategy exists to date. Second, building on the Expert Panel work and consolidating advice and work from consultations from a national health literacy advisory group after two think tanks and a workshop, in 2012 an *Intersectoral Approach for Improving Health Literacy for Canadians* (Action Plan) was released [19]. The development of the Action Plan is an approach to promote health literacy and inform policy across provinces/territories [49]. After all, this Action Plan it is not official policy. The Action Plan identifies three fundamental components (develop knowledge; raise awareness and build capacity; build infrastructure; and partnerships) and five settings (governments; health sector; education sector; workplaces and businesses; community organizations) deemed essential in a strategy for improving health literacy in the population [19]. The 2008 Expert Panel report noted that a lack of awareness about health literacy was impeding efforts and that a survey of nearly 700 professionals and policy-makers found that almost 60% indicated the staff in their organizations did not know where to find resources to support health literacy efforts. Despite the 2012 Action Plan as a step-in right direction towards a promising remedy, the question remains: How much has changed without formal national policy, mandate and associated funding to incorporate health literacy into Canadian efforts since 2012?

### 3.2. Policy in the United States

In 2010 in the United States, there were three major federal-level policy initiatives and laws related to health literacy that collectively were implemented to help address the low levels of limited health literacy across the country. These include the Affordable Care Act of 2010, the Plain Writing Act of 2010 and the 2010 National Action Plan to Improve Health Literacy.

The Affordable Care Act (ACA) of 2010 (nicknamed Obamacare) was signed into law by President Obama on 23 March 2019 [55]. According to a 2010 Institute of Medicine health literacy paper, this law has direct and indirect links to health literacy in the following six health and health care domains [56]: (1) coverage expansion (e.g., by enrolling, reaching out to and delivering care to health insurance coverage expanding populations); (2) assuring equity (e.g., in health and health care for all communities and populations); (3) workforce (e.g., training providers on cultural competency, language, and literacy issues); (4) patient information (e.g., appropriate reading levels); (5) public health and wellness; and (6) quality improvement (e.g., more efficient models of care with those with chronic illness and requirement self-management).

The Plain Writing Act of 2010 is a law requiring federal agencies to use clear communication that the public can read, understand and use. The intent of the law is to help people not only apply and use a range of health-related materials, but also apply for the range of services and benefits for which they are eligible beyond the health sector [57].

In 2010, the US Department of Health and Human Services [9] produced *The US Action Plan to Improve Health Literacy* that calls for a multi-sectoral effort to improve health literacy presenting seven goals and associated strategies for the country: (1) health information creation and dissemination; (2) health care services; (3) early childhood through university education; (4) community-based services; (5) partnership and collaboration; (6) research and evaluation; and (7) dissemination of evidence-based practice. The US Action Plan provides a framework and when individuals, institutions, or organizations ask what they should be focusing on in their educational practice they are pointed to Goal 3. This goal area highlights early childhood, K-12, and higher education to designate students and learners as agents of change to promote health literacy and improve health and learning.

Other notable developments in the US are national standards and objectives that support these federal health literacy policies. For example, the seven National Health Education Standards (NHES) were originally crafted in 1995 considering characteristics of a health-literate individual in the broad context of health and being essential to health literacy [58]. The revised 2007 NHES contain 8 standards that provide a framework and continue emphasizing ‘teaching to the skills’. The NHES are intended to align curriculum, instruction, and assessment K-12 [59]. Moreover, the revised National Standards on Culturally and Linguistically Appropriate Services (CLAS) Standards consist of 15 action steps for all individuals and organizations intended to help eliminate health care disparities and advance health equity to implement culturally and linguistically appropriate services [60]. The Healthy People 2020 and 2030 [61] includes an objective to achieve health literacy.

### 3.3. Policy in Germany

In Germany, which is a federated country, health literacy has recently been considered an important topic for policy making. There are currently three national-level policies, of which one is binding and represents an enacted law, while of the other two, one is a policy initiative of a network of health care partners and the other represents an action plan with recommended action. These three policy initiatives are: (1) the law on strengthening health promotion and prevention (Prevention Law) [62] (2) the national health literacy alliance [63], and (3) the German National Action Plan on Health Literacy [64].

The Prevention Law represents a binding policy and was enacted in 2015 after more than ten years of development and several setbacks. By law, this policy document requires various actors on the national and state level to take responsibility to define and implement action on health promotion and prevention. Among these actors are the social insurance and security agencies, including statutory health insurance funds, pension funds, employer’s liability and accident insurance associations, and statutory nursing care insurance that covers home care, health care, and as the short and long-term care needs of citizens. Further agencies, institutions and organization involved in health promotion and prevention are also addressed by this law, such as state family counselling, care and aid agencies as well as the Federal Centre for Health Education (BZgA).

On the behavioral level, the Prevention Law calls for strengthening the responsibility and competencies of citizens to promote their health and participate in preventive action. The law mentions health literacy, specifically increasing the health literacy capacities of families. It also describes measures to support and invest in children’s education in and outside family homes, including the consideration of family socioeconomic situations in the context of interventions. The importance of increasing the uptake of help services and systems, and strengthening family cooperation with government and state organizations and departments [62] (p. 1375; 52, Section 16 SGB VII). Other sections of the law describing health promotion dimensions use synonyms for health literacy, e.g., ‘health-related life-skills,’ ‘health skills and competencies,’ and ‘skills to promote health behaviors’ [61] (p. 1369). This mandatory policy is supposed to be implemented on national and state levels. Some states have also developed their own policies based on the Prevention Law.

The German Alliance for Health Literacy is a policy initiative launched in 2017 by the Federal Ministry of Health in cooperation with various stakeholders from the health care sector [63]. The overall aim of this policy is to develop and implement a nation-wide and sustainable strategy to strengthen health literacy in the general population in Germany. This policy outlines a community and citizens-based strategy addressing local living environments and includes a two-pronged approach: (1) accelerating, strengthening, and coordinating existing programs, measures, and approaches of health care sector organizations to strengthen health literacy; (2) developing and implementing new programs, measures and, approaches based on best practice examples and international standards to strengthen health literacy. Main partners in this initiative are the Federal Ministry of Health, the national umbrella organizations of the health care sector, which represent different high-level confederations and include more than two dozen public health, medical, health care, nursing and insurance associations, and the members of the national health conference, including the state health ministers and senators. Further civic and socio-political organizations and associations, i.e., from the education and social sectors are supposed to be integrated in the Alliance initiative. The immediate goals of this policy are: (1) to strengthen population health literacy through health education in all settings; (2) provide evidence-base health information and services in easy and plain language, especially on the internet; and (3) address health communication in training and education of health professionals.

The German National Action Plan on Health Literacy [64] (Action Plan) is a non-binding policy document developed by civil society actors and experts from health care, public health, medicine, and education supported by the Federal Ministry of Health. The Action Plan can be considered as a guideline document similar to action plans of other countries such as the Canadian and US action plans. It defines several action areas, implementation principles and recommendations to strengthen health literacy in the German population, in organizations, on the systems-level, and in the policy sector. The Action Plan understands that health literacy needs to be addressed within a whole-of-society approach including, the policy sector, health care sector, education sector, research sector, workplaces, professional training as well as communication and consumption. In particular, The Action Plan embeds health literacy into a greater health promotion and prevention strategy aiming at action in everyday life environments, in the context of chronic disease and all levels of the health care system. Research complements the strategy and is critical to generate evidence to inform the outlined action and thus needs appropriate support by the policy sector specially to ensure funding. The implementation principles include to: (1) facilitate collaboration between all actors; (2) use potentials of digitalization for health; (3) reduce social and health inequalities; (4) enable and increase participation; and (5) address both individual health literacy and environmental and organizational health literacy. Embedded into this framework, the Action Plan highlights 15 recommendations to address health literacy challenges in the contemporary German society. The first recommendation specifically focuses on the education sector and enabling the education system to promote health literacy early in life. The plan was published and introduced in the Federal Ministry of Health in Berlin in 2018.Currently the second funding period is being planned aiming to: (1) implement the plan and (2) develop state action plans on health literacy to ensure the uptake on the state government-levels in the sixteen federal states. The second action plan cycle will be coordinated by the Bielefeld University’s Centre for Interdisciplinary Health Literacy Research and most likely will start sometimes in 2020.

In summary, policy-wise Germany seems to be well equipped to strengthen health literacy through the educational system, but these policies are rather new, and only time will tell whether they will be implemented and backed by funding, financing, and other resources. Although all three policies highlight the relevance of the education sector in order to strengthen health literacy skills from a young age onwards, the education sector itself still has not included health literacy as an agenda item in their own policies, which is why the implementation of health literacy in schools in Germany is not where it should be at this time [29,30].

## 4. Gaps and Tensions

Table 1 provides helpful foundational educational context and comparison of educational structures in Germany, Canada, and USA with relevance for the education for health literacy perspective [65,66,67]. While we believe the *education for health literacy* perspective is a useful paradigm and should be pursued in policy and practice, there are gaps and tensions. We consider several of these following Table 1.

### 4.1. Health Literacy Definitions

Healthy literacy has a definition, but there are many definitions. The same is true of policy. This has implications for research and practice; operationalization and prioritization.

(a) Health literacy means different things to different people. This makes measuring (or even talking about) the concept, process, and outcome challenging.
(b) Policy means different things to different people. There is policy, public policy, health policy, and health literacy policy. There does not seem to be a consensus on health literacy policy. While we use the aforementioned definition for policy in the paper, and do think policies in the broader definition are relevant to momentum and conversations around topics (in this case health literacy), many of the health literacy policies are non-mandatory and non-binding. Instead, they are promoted and recommended. These can be powerful and influential (e.g., WHO Shanghai declaration as a good example), but may not actually be prioritized or implemented in given competing priorities and funding constraints in practice if they are not mandatory.

### 4.2. Equity

The educational system should provide a pathway to basic/functional literacy as well as communicative/interactive and critical literacy. Yet in some school districts in the US, even the most basic, functional literacy skills are not achieved [68]. In Canada, there are educational barriers faced by some Aboriginal peoples, specifically related to postsecondary educational opportunities due to historical, geographical, cultural, and individual barriers [69,70]. Thus, these populations do not achieve literacy despite their years of schooling, or may obtain unequal literacy despite the same years of schooling [71]. Possibly related to this, the impacts of education on health appear to be less for minorities, and minorities report poorer health status compared to whites at every level of educational attainment [72,73].

There are strategies to help improve educational and health outcomes for Indigenous peoples in Canada. Embedding education for health literacy supporting Aboriginal identity through Aboriginal elders using their own languages and cultural methods of teaching and learning is crucial, while addressing the identified barriers related to the other social determinants of health to improve health and learning outcomes. National educational policies in the US, sometimes spurned by lawsuits and activism, attempt to address these inequities, but these issues are important to consider in achieving the promise of the education for health literacy paradigm across educational sectors. This may also be an opportunity for adult education to address. Educational attainment impacts individuals, but also health across generations; parental education is associated with children’s health and children’s educational attainment [74]. Generations of educational inequity can perpetuate health inequalities over time. We should prioritize their elimination.

### 4.3. Theory to Mandated Practice

Health literacy actions and activities may be informed by evidence, theory, models and frameworks, but the question that arises if how far does it really get into practice. Implementation can reveal challenges, gaps, and tensions resulting in practice variation and differential success. Below are some factors that produce gaps and tensions:

(a) States adapt from national models with their own interpretation, which can lead to idiosyncratic models and fragmented outcomes.
(b) No funding attached.
(c) Mandating does not mean implementation will occur.
(d) Even if mandated, human capacity requirements (e.g., trained staff, improved education) are often lacking to ensure operationalization and sustainability.
(e) Competing priorities in education system.
(f) Oversight around implementation is often minimal.

### 4.4. Evaluation and Cost Effectiveness

The evidence that these policies matter for health and the results of programs are often not evaluated, particularly not in terms of cost effectiveness.

(a) Follow up is often not done. No evaluations. No evidence reviews.
(b) Evidence is about literacy or health education, but not “health literacy”.
(c) Action plans exist, but is there evidence to back these “recommendations”.

### 4.5. Health vs. Education

Health system goals dominate education goals (and funding).

(a) Fragmented training of teachers in the US and Canada, coupled with the decentralized system make it difficult to ensure a quality national education-related effort even if there is a policy manifest.
(b) Whole school and healthy school approaches and premises may be preached in countries, but in reality, the health and education link is not fully operationalized. Health and education sectors are not coordinating as well as they could due to system goals.

## 5. Strategic Directions and Opportunities

This study points to a variety of future directions for research and practice. The relationships of education and health and health literacy may change over time. For instance, older adults may have cohort-specific educational experiences. Deeper considerations of the role of education outside the formal educational sector would be useful, especially models that consider health literacy from education to home to other aspects of one’s life and how one may integrate this into their life would be useful.

Despite the tensions and gaps in the education for health literacy approach and the limitations of scope from our Perspective Piece primarily on the formal educational sector, we end on a promising note. This section demonstrates innovative models in the education sector using examples from Canada. Given this country’s recognition as one global leader of practice in increasing the health literacy capacity of individuals, providers, and systems with partnerships coupled with its positive PISA scores (see Table 1), we choose to highlight selected noteworthy education for health literacy examples from this country. These are not all within the formal educational sector.

### 5.1. Canadian Pockets of Practice

In 2011, the University of British Columbia the Public Health Agency of Canada identified noteworthy health literacy initiatives across Canada. Working with the Canadian Public Health Agency [63], examples were featured in an online document *Examples of Health Literacy in Practice*. Examples were further condensed based on inclusion and exclusion criteria and can be accessed on the website of the Canadian Public Health Agency [75]. This resource of peer nominated examples of flagship health literacy initiatives covers a range of target groups, settings, and health topics in different provinces/territories. In the educational resource, “a variety of approaches to delivering health literacy information and services are featured including courses and workshops; patient-provider communication; prevention and treatment; peer navigation; information development and dissemination; and community development” [75] (p. 7). This particular national resource reiterates that ‘education for health literacy’ is necessary for both those who access and those who provide health information, programs and services. Appendix A depicts additional health literacy practice examples that focus on the education perspective and highlight the need and benefit for action. Educational activities and initiatives are in the forefront in these examples in building health literacy knowledge and skills across the life-course.

### 5.2. Future for Education for Health Literacy

Reflecting on the snapshot of diverse ways in which the education for health literacy perspective can be found in our national health literacy policies, followed by some tensions and gaps coupled noteworthy examples that have surfaced during the translation and implementation of these policies, we consider key questions to move forward. What is our ideal future for education for health literacy in Canada, US, Germany and elsewhere? Are there common denominators? What do we need to succeed to get there?

A universal goal is to achieve equitable levels of health literacy for all through our policies and practice in a supportive environment. From both a contextual and practical view, advancing education for health literacy for each nation requires emphasizing it as a process starting early in life and recognition of it as an essential daily resource for the life-course.

For each nation, education for health literacy involves a recommended set of actions framed within each country’s respective national health literacy action plan that could be taken at the national, provincial/territorial, state and/or local levels with the purpose of enhancing health literacy knowledge and skills. In a binding policy, as seen in the US National Health Literacy Action Plan document, we see that policy can inform practice. In a non-binding policy document, as seen both in Canada’s and Germany’ health literacy action plans, we can see that practice can support non-binding policy. Noteworthy health literacy practice initiatives can be a catalyst for recognition among governments that groundwork has been sufficiently laid and provide promise for policy development [49].

To further advance and succeed in education for health literacy efforts, we need both policy and practice champions. Frankish [76] has identified several factors that influence the advancement of promising health literacy work. Key success factors relate to addressing the often limited capacity and number of trained people to do and measure this work coupled with lack of funding and leadership [75]. The education for health literacy perspective promotes intersectoral collaboration and partnerships casting a net for greater success for health literacy initiatives, increasing innovation among different team thinkers, and pooling the health literacy tools thus, avoiding duplication of resources resulting in better education and health outcomes.

## 6. Conclusions

As summarized by the recent Position Statement (Statement) put forth by the International Union for Health Promotion and Education (IUHPE) Health Literacy Global Working Group [6], “health literacy is an asset that can support a wide range of health actions to improve health and well-being, to prevent and better manage ill-health” and can help us achieve health equity. We believe the education sector is critical to this goal. This article provides landscaping, practical guidance, an eye to gaps and tensions towards this goal from three countries that have each played important roles in global health literacy research, policy, and practice.

## Figures and Tables

**Table 1:** Comparison of educational structures in Germany, Canada, and USA with relevance for the education for health literacy perspective.

	Germany	Canada	USA
**K-12 System Structure**	Federal system; academic year runs Fall—Summer (e.g., October–August)	Provincial/territorial system with provinces (10) and territories (3) having autonomy; academic year runs Fall—Spring (e.g., September–June)	Federal system with strong role for local governments; academic year (Fall—Spring (e.g., August–June)
**K-12 School Governance**	Mostly controlled by the 16 Länder (states)	Most guidelines set at the province and territory level.	Mostly in control of local school boards.
**Higher Education System Structure**	Federal academic education framework available, but due to the national higher education act, the sixteen state governments have the responsibility for design, content and aims of their universities. However, to certain degree the state and federal government(s) collaborate.	Provincial/territorial system is responsible for education and regulate standards; no federal accreditation; academic year split into 3 semesters [Fall (end of Aug/start of Sept to December), Spring (January to April), Summer (April/May to July/August)]	Federal and state systems responsible. Accreditation involves non-governmental entities as well as federal and state government agencies. Accreditation’s quality assurance function is one of the three main elements of oversight governing the Higher Education Act’s (HEA’s) federal student aid programs.
**Higher Education Governance**	Internal with university president, team of administrative leaders (provost, chancellor, faculty senate, deans, department chairs)	Internal with university president, team of administrative leaders (provost, chancellor, faculty senate, deans, department chairs)	Internal with university president, team of administrative leaders (provost, chancellor, faculty senate, deans, department chairs)
**Testing**	Secondary schools in Germany are divided among several tiers. Cumulative test to leave school.	Similar to US, almost all provinces conduct standardized tests from junior kindergarten into high school (e.g., reading, writing, math, science), but these vary by province.	Since 2002, there is a requirement for testing of all students in middle grades and sometimes in high school. These tests are standardized by state. Only basic skills test required to complete school.
**Teacher Training**	Standards for teacher education are centralized within each state with some coordination across states.	Standards for teacher education Teaching is selective in Canada.	Teacher training is decentralized, state requirements vary widely, and oversight can be lax.
**Teacher Pay**	Most teachers are civil servants and are paid well (relative to their US counterparts).	Teachers are paid well (relative to their US counterparts).	Teachers are paid poorly.
**Curriculum Consistency**	Curriculum recommendations and frameworks available provided by the Kultusministerkonferenz (KMK) but, due to the federated system the states modify those based on their needs and policies.	Much consistency across schools and districts in curriculum and teaching methods.	Little consistency in training, curriculum, or methods.
**Federal Government Focus**	Health equity and health inequalities in several policies and the health promotion law.	A strong common commitment to equity across provinces/territories	Compensatory education for students from low-income backgrounds and special education for students with disabilities.
**Equity**	In general whole-of-society, but families, special needs (“inclusion”) and Immigrant and refugee communities are a focus for equity efforts. (idealistic; implementation of equity action is not secured)	Focus on at risk youth, migrant, and indigenous populations. Canada has many immigrant families, but is one of the few countries where migrant children achieve at a level similar to their non-migrant counterparts.	Focus on racial/ethnic minorities and urban inequities. While the federal government’s role has focused on ensuring equity for disadvantaged populations, deep inequities by race/ethnicity remain.
**Groups of Focus for Equity**	Migrants, students with disability and special needs (“inclusion”), to a certain degree all students are supposed to benefit from equity action in schools. (idealistic; implementation of equity action is not secured; Germany has a multi-track school system in place starting with secondary school and which might contribute to inequities and inequalities by its very structure as at the end of primary school pupils are being distinguished by their marks, which determine which school they can attend within this multi-track system.)	Canada has many immigrant families, but is one of the few countries where migrant children achieve academic outcomes at a level similar to their non-migrant counterparts.	Compensatory education for students from low-income backgrounds and special education for students with disabilities.
**How Health Literacy is Addressed**	No formal, national health literacy standards available. Guidelines exists (e.g., Digital education standards; Life-skills in the school health promotion strategy)	No formal, national health literacy standards available. Guidelines exists (e.g., School Health Guidelines, Sexual Health Education Guidelines)	National Health Education Standards: Achieving Health Literacy; Achieving Excellence (NHES, 1995; 2007)
**Whole School Approach**	No mandatory whole-school approach, but a modified Health Promoting School approach is in some regions in place, where participation of schools is not mandatory. Health literacy is not yet part of this.	No mandatory whole-school approach, but many provinces have a website for their own Healthy Schools approach with resources. Provinces vary with their framework used. Extent to which health literacy is a part of this varies across provinces.	Whole School, Whole Community, Whole Child approach promoted nationally; extent to which this is utilized varies across states. Health literacy is embedded in this.
Pisa Score Science *	509	496	528
**Pisa Score Reading**	509	497	527
**Pisa Score Math**	506	470	516
**Pisa Share of Top Performers in At Least One Subject Science, Reading and Mathematics**	19.2	13.3	22.7
**Pisa Share of Low Achievers in All Three Subjects (Below Level 2)**	9.8	13.6	5.9

Note: Benchmark scores for low performance can be found http://www.oecd.org/pisa/pisa-2015-results-in-focus.pdf [67].

## Appendix A

Canadian examples: Education for health literacy across the life course.

**Across the** **Life Course**	**Non-Profit Organization**	The Indigenous Story Studio (formerly the Healthy Aboriginal Network) is a non-profit organization based in British Columbia whose purpose is to create entertaining knowledge translation tools for youth (pre-teens to early 20 s) on health and social issues using modalities such as comic books, graphic novels, film/video, animation and augmented reality, all of which take into consideration providing youth with information that they can understand and that appeals to them. For more information visit: https://istorystudio.com/about-us/
	**University Course**	In 2013, Vamos developed and currently teaches the first Canadian core undergraduate health literacy course titled *Health Literacy and Systems Navigation* in the School of Public Health & Social Policy at the University of Victoria in British Columbia [24]. Aligned with the aforementioned Canada’s milestone reports that promote ‘education for health literacy’ focusing on higher education, this innovative course provides future allied-health professionals the opportunity to explore practices, tools, and policies guiding health literacy efforts for diverse people across settings and the life-course. It was recently adapted and used as a blueprint for a proposed introductory online European health literacy course for two German universities [77].
	**Provincial Network**	The BC Health Literacy Network is a unique network of networks of people and organizations representing various sectors working together (e.g., education, health care, library, seniors, government) committed to the advancement of health literacy. Since its establishment in 2011, Rootman continues to lead the steering committee meeting monthly to pursue goals articulated in a provincial strategy [78] and to help organize educational activities related to the goals. Example activities include: a two-day summer school; community of practice meetings; webinars; and workshops to raise awareness and build capacity of the BC networks to deliver health literacy programs over the life-course.
	**Provincial Organization**	The Council of Senior Citizens Organization (COSCO) of British Columbia [79] is an umbrella organization made up of seniors’ organizations and individual associate members representing about 80,000 seniors. A grant from the former Canadian Council on Learning led to the development of a series of workshops related to health literacy using a “training of trainers” model. Currently, on request, they offer workshops on 43 different issues and topics of particular interest to seniors at no cost, delivered mostly by seniors trained to do so using “plain language” principles. For more information visit: http://www.coscobc.org/index.php/cosco-workshops
